# In Vivo ORF Overexpression Screening Identifies CCN4 as a Regulator of Glioblastoma Growth Validated Across Multiple Models

**DOI:** 10.3390/ijms27125227

**Published:** 2026-06-09

**Authors:** Pushan Dasgupta

**Affiliations:** 1 Department of Genomic Medicine, UT MD Anderson Cancer Center, Houston, TX 77030, USA; pushan.dasgupta@bcm.edu; 2 MD Anderson UTHealth Houston Graduate School of Biomedical Sciences, Houston, TX 77030, USA; 3 Division of Neuro-Oncology, Department of Neurology, Baylor College of Medicine, Houston, TX 77030, USA; 4 Dan L. Duncan Comprehensive Cancer Center, Baylor College of Medicine, Houston, TX 77054, USA

**Keywords:** glioblastoma, functional genomics, in vivo screen, overexpressed driver, CCN4, glioma, high-grade glioma

## Abstract

Despite current multimodal therapies for glioblastoma (GBM), its prognosis remains grim. Thus, a tremendous need exists to identify new genetic drivers that may serve as potential therapeutic targets in glioblastoma (GBM). We describe an in vivo overexpression screening strategy to identify drivers of glioblastoma where we have leveraged TCGA datasets to conduct a functional genomics screen of prioritized open reading frames (ORFs) that are overexpressed and/or amplified in GBM. To interrogate these potential drivers within a more relevant physiological context, the screening was accomplished in vivo in an orthotopic patient-derived glioma stem-like cell (GSC) model. Among 5 positive “hits” from the screen, Cellular Communication Network factor 4 (CCN4) was prioritized for further evaluation. Our functional analyses demonstrated that CCN4 overexpression drives tumor growth in multiple GBM models. Depletion of CCN4 reduced growth in vitro and in vivo and markedly decreased colony formation with the growth phenotype restored upon ectopic expression of CCN4. Structural functional analysis of CCN4 was also conducted. We believe that this screening strategy can serve as a platform for further identification and validation of drivers of GBM.

## 1. Introduction

Glioblastoma multiforme (GBM) is the most common and most malignant primary central nervous system tumor [1,2,3,4,5]. Uncontrolled cellular proliferation, widespread invasion throughout the brain, profound angiogenesis, and a proclivity for necrosis are just some of its key characteristics [6]. Patients who receive current standard of care, which entails neurosurgery and subsequent concomitant chemoradiotherapy followed by systemic temozolomide in the adjuvant setting [1], achieve median survival of less than 15 months, and less than 5% of patients survive longer than 3 years [7]. A tremendous need exists to find new therapies to improve the prognosis for patients with these highly recalcitrant tumors.

Multiplatform studies like those conducted by The Cancer Genome Atlas (TCGA) Research Network consortium have analyzed the genomic and transcriptomic landscape in tumor tissue samples from hundreds of patients with untreated primary GBM [8,9]. One way to leverage this wealth of data is to design functional genomic screens aimed at identifying and defining drivers of glioblastoma. Here we report an in vivo functional overexpression screening strategy that utilizes established patient-derived glioma stem-like cells [10] to interrogate a gain-of-function library of genetic elements of interest generated by analyzing the TCGA glioblastoma dataset. Using this screening strategy we have been able to further validate Cellular Communication Network factor 4 (CCN4, formerly known as WNT1-inducible signaling pathway protein 1 [WISP1]), a protein known to be a secreted component of the extracellular matrix, as a driver of GBM [11,12,13]. We believe that this screening strategy can serve as a platform for further identification and validation of drivers of GBM.

## 2. Results

### 2.1. In Vivo Context-Specific Gain-of-Function Overexpression Screen

**Figure 1:**
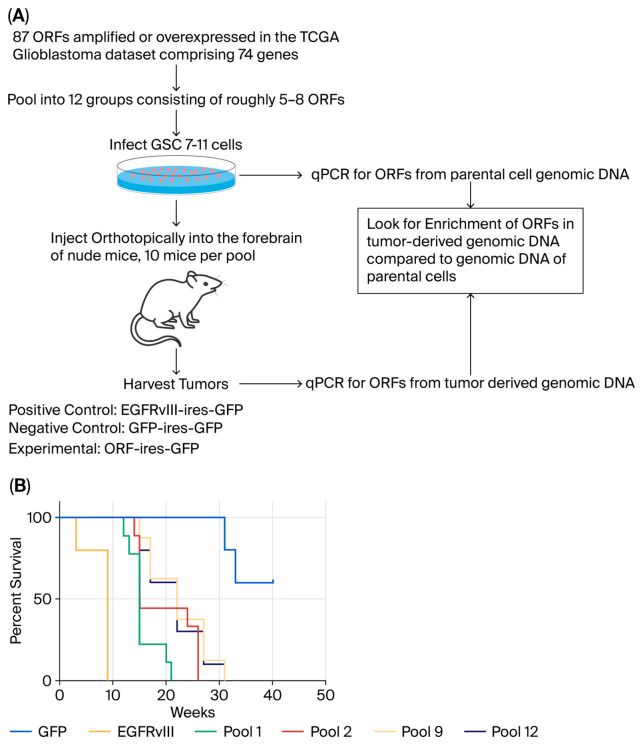
Overexpression gain-of-function screen. (**A**) Screen Schema (**B**) Survival curves for the pools from the screen that had a reduced latency. LogRank (Mantel Cox) test shows statistical significance for the survival of each pool relative to the GFP negative control: Pool 1 (n = 9) *p* = 0.0005, Pool 2 (n = 9) *p* = 0.0009, Pool 9 (n = 8) *p* = 0.0018, Pool 12 (n = 10) *p* = 0.001), n = 5 for GFP control. *p*-values less than 0.05 were considered to be statistically significant.

We queried the GBM TCGA datasets to produce a list of 87 open reading frames (ORFs; 74 genes) frequently amplified and/or overexpressed in GBM (Supplemental Table S1), as we reasoned these represented candidate drivers of the disease. To maintain transduction efficiency, we chose the list based on ORFs with the smallest nucleotide size. To enable the detection of reduced latency of tumor development, we selected a relatively slow-growing GBM cell line model established from patient-derived glioma stem-like cells (GSC), GSC 7-11 ([14], Figure 1A). Cells were infected with a pool of 5- 8 lentiviral constructs at a multiplicity of infection of 3. ORFs were ordered from smallest to largest with each pool having similar sized ORFs to make sure the transduction efficiency was similar in each pool for each ORF. An aliquot of the infected cells from each pool was stored as a reference sample, and the remaining cells were injected orthotopically into the forebrains of nude mice and their growth was monitored over time. Animals injected with cells transduced with either epidermal growth factor receptor variant III (EGFRvIII) or green fluorescent protein (GFP) constructs were included as positive and negative controls, respectively, to compare tumor latency periods [15].

**Figure 2:**
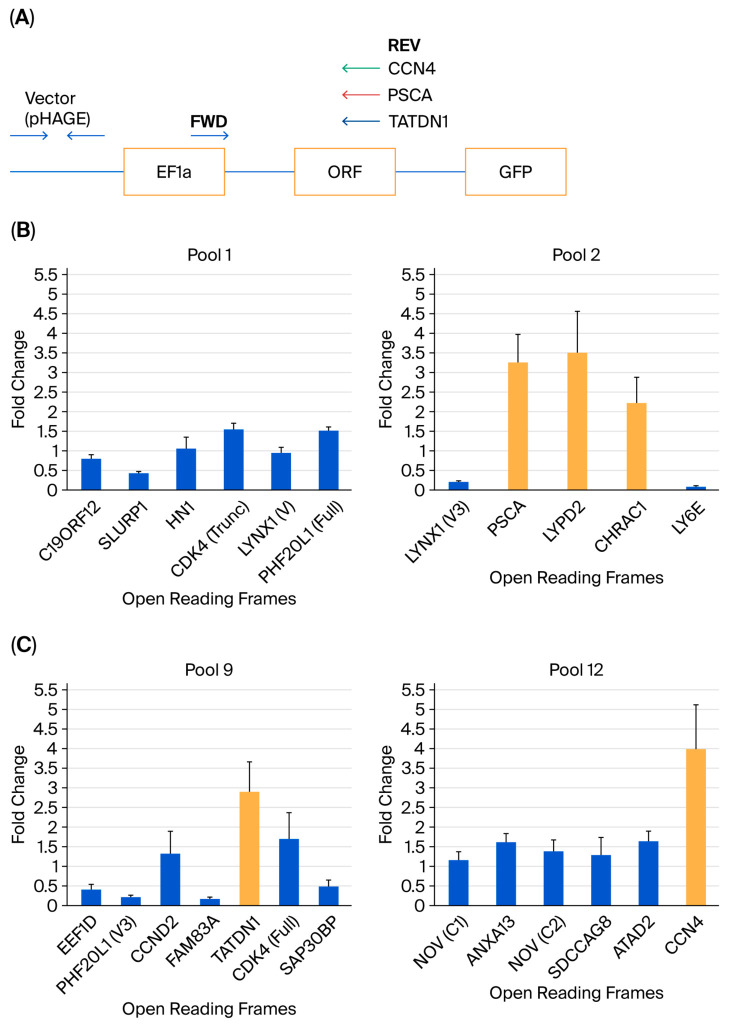
qPCR strategy and analysis of screen results. (**A**) Diagram depicting the qPCR strategy. Detailed construct in Supplemental Figure S5. (**B**,**C**) For pools with reduced latency, qPCR fold changes are shown for each ORF relative to the reference. The mean represents the average fold change for each ORF from 5 tumors derived from the first 5 mice in each pool to be sacrificed. (**B**) Pools 1 and 2 (**C**) Pools 9 and 12. Error bars represent the standard error of the mean. ORFs are also specified in parentheses as either: TRUNC = truncated, V = variants, Full = full length, C = ORF clones. Genes with ≥2-fold change are indicated in yellow.

Among the 12 cohorts of mice injected with infected tumor cells, mice harboring cells infected with ORF pools 1, 2, 9, and 12 developed tumors more rapidly compared to the GFP control group (Pool 1 (n = 9) *p* = 0.0005, Pool 2 (n = 9) *p* = 0.0009, Pool 9 (n = 8) *p* = 0.0018, Pool 12 (n = 10) *p* = 0.001), n = 5 for GFP control) (Figure 1B). Tumors from these cohorts were harvested and genomic DNA was extracted for analysis compared with genomic DNA from the reference cells. Representation of ORFs was quantified via qPCR normalized to the amplification values generated on a non-variable region of the plasmid (Figure 2A). We hypothesized that cells transduced to overexpress a driver would begin to dominate the population of cells in the tumors with reduced latency such that qPCR of genomic DNA of the tumor compared to that of the parental cells would indicate significant fold enrichment for the driver gene. In our qPCR strategy the forward primer was the same for all the ORFs as it was complementary to a portion of the EF1-α promoter but the reverse primer was unique to each ORF. For normalization two primers outside the long terminal repeat (LTR) sequences were used to amplify a portion of the plasmid which served as the control (Figure 2A).

To prioritize genes for validation, we first selected genes for which a 2-fold or greater enrichment compared with reference cells was observed. In pool 1, none of the candidates were enriched to this extent. Among the others, the 2-fold enrichment cutoff identified five candidate driver ORFs that we decided to subject to further analysis: prostate stem cell antigen (PSCA), Ly6/PLAUR Domain Containing 2 (LYPD2), chromatin accessibility complex 1 (CHRAC1), TatD Dnase Domain Containing 1 (TATDN1), and Cellular Communication Network factor 4 (CCN4) (Figure 2B,C). To better understand whether these candidates represented ORFs that may have clinical significance in glioma we conducted analysis using data from an independent database not related to the TCGA. We therefore used the Chinese Glioma Genome Atlas (CGGA) [16]. We were able to find data on all the candidates except LYPD2 in the CGGA for analysis. Among the candidates, higher grade of glioma correlated with higher expression of CCN4 and CHRAC1, and this was statistically significant in both cases (Supplemental Figure S1A,B). Patients with higher CCN4 expression had worse overall survival which was statistically significant (Supplemental Figure S1C). Patients with higher expression of CHRAC1 and PSCA demonstrated a trend toward worse overall survival; however, this difference did not reach statistical significance (*p* = 0.24, *p* = 0.16, respectively), likely reflecting limited cohort size (Supplemental Figure S1D,E).

In our screen CCN4 had the highest fold change and stood out in Pool 12 (Figure 2C). There is growing evidence that CCN4 is involved in GSC maintenance and may serve as a driver of glioblastoma [12,13]. To corroborate those findings and validate this screening strategy we prioritized CCN4 for functional validation using our GSC and GBM lines.

### 2.2. CCN4 Drives Tumor Growth in GBM

**Figure 3:**
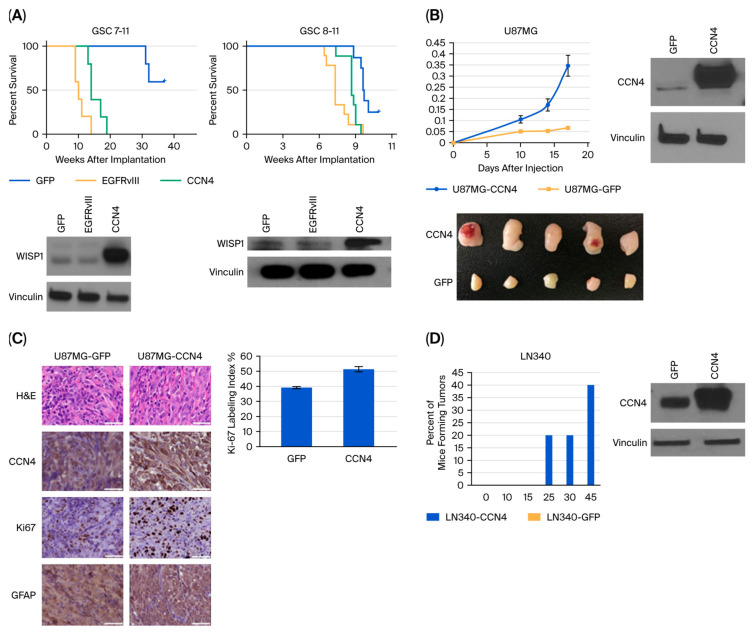
CCN4 overexpression drives tumor growth in vivo in GBM models. (**A**) Survival curves for mice orthotopically injected with transduced GSC 7-11 cells (left) or GSC 8-11 cells (right). For GSC 7-11, n = 5; for GSC 8-11, n = 9 (CCN4) and n = 8 (GFP). Tumor latency was compared using the LogRank (Mantel-Cox) test. GSC 7-11, *p* = 0.0005; GSC 8-11, *p* = 0.0018 based on LogRank (Mantel-Cox) test. The associated immunoblots are shown below for the respective GSC line. (**B**) Tumor growth after subcutaneous injection with U87MG cells transduced to overexpress CCN4 or GFP (n = 10 mice/group). Error bars represent the standard error of the mean. 3 million cells were injected per mouse. Photo is of tumors at the final time point. Respective immunoblot is to the right. (**C**) Immunohistochemistry of tumors derived from U87MG cells. Scale bar, 25 μm. The Ki67 labeling index was measured for both groups. The error bars represent standard deviation. *p* = 0.0001 based on two-sided *t*-test. (**D**) Percent of tumor formation over time after subcutaneous injection with LN340 cells transduced to overexpress CCN4 or GFP (n = 10 mice/group). 3 million cells were injected per mouse. Respective immunoblot to the right. *p*-values less than 0.05 were considered to be statistically significant.

We transduced the patient-derived glioma stem-like cells used in the in vivo screen, GSC 7-11, as well as a second one, GSC 8-11, with a CCN4-expressing construct driven by the EF1-α promoter, injected them in the forebrain, and measured tumor growth over time. As positive and negative controls, respectively, we utilized EGFRvIII and GFP-encoding lentiviral constructs. CCN4 overexpression resulted in reduced latency and survival in both models (GSC 7-11, *p* = 0.0005; GSC 8-11, *p* = 0.0018) (Figure 3A). In order to see if the phenotype was dependent on the brain-specific tumor microenvironment we wanted to see if CCN4 overexpression could accelerate tumor growth in in xenograft models with injections to the mouse flank. We therefore used established GBM models. Similarly, CCN4 accelerated tumor growth and reduced animal survival in an established GBM model, U87MG (Figure 3B). Immunohistochemistry of these tumors demonstrated that they were astrocytic and Ki67 labeling index was higher in the CCN4 overexpressing tumors (*p* = 0.0001) indicating increased growth (Figure 3C). Significantly, ectopic overexpression of CCN4 induced tumor formation in LN340, an established glioblastoma cell line that grows poorly in vivo. Specifically, 40% of animals harboring CCN4-overexpressing LN340 cells developed tumors compared to none developing tumors in the GFP-expressing control group (Figure 3D). Immunohistochemistry was performed on these tumors as well (Supplemental Figure S2). Western blot was used to verify overexpression of CCN4 in all cases (Figure 3A,B,D).

**Figure 4:**
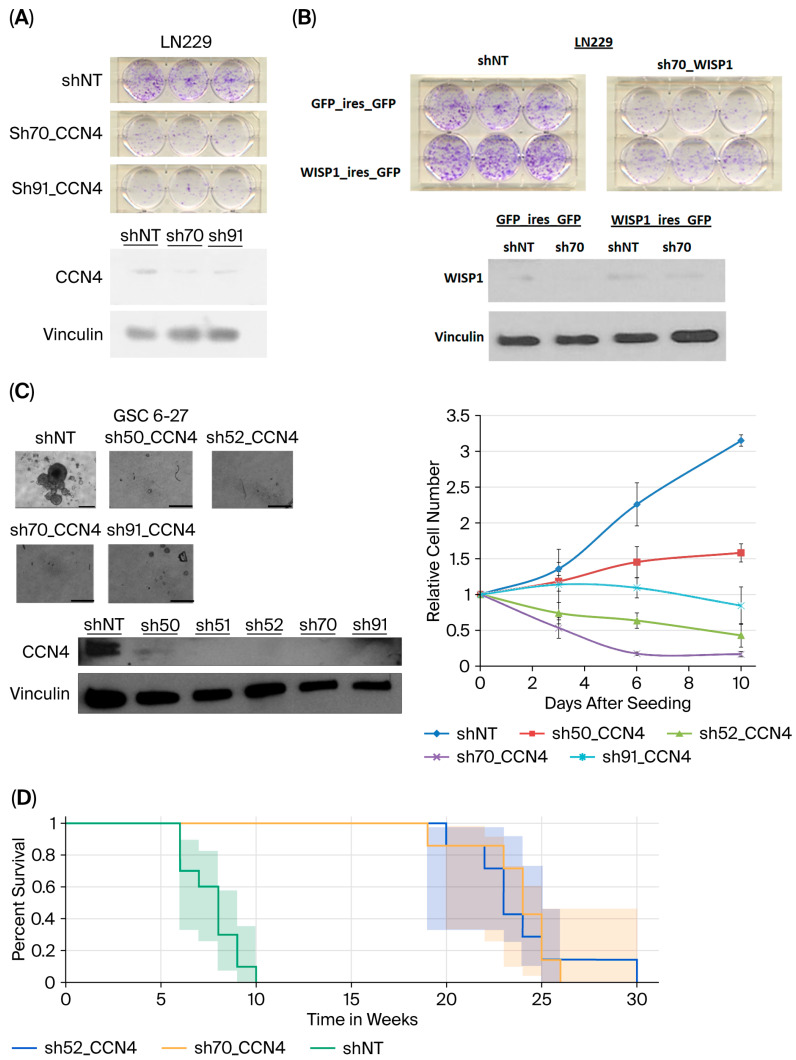
CCN4 knockdown reduces growth in vitro in GBM lines. (**A**) Colony formation assay in LN229 after CCN4 knockdown with two independent shRNAs. Immunoblot confirms knockdown of CCN4 by shRNA (sh70, sh91) compared to non-targeting shRNA (shNT). (**B**) Rescue experiment in LN229 cells expressing either pHAGE-CCN4_ires_GFP or pHAGE-GFP_ires_GFP. The cells also expressed either an shRNA targeting CCN4 (sh70) or a control shRNA (shNT). Protein expression is shown in the immunoblot for each condition. (**C**) Growth of GSC 6-27 cells in culture after knockdown of CCN4. Values are relative cell growth based on CellTiterGlo measurements of viability over time normalized to day 0. Error bars represent standard error of the mean. Images taken at final time point. Scale bar, 100 μm. Immunoblots for CCN4 in control and knockdown lines are also shown. (**D**) Survival curve for mice orthotopically injected with GSC 6-27 cells transduced with shNT control n = 10, sh52_CCN4 n = 10, sh70_CCN4 n = 10. *p*-value: 0.000052, based on LogRank (Mantel-Cox) test. *p*-values less than 0.05 were considered to be statistically significant.

To determine whether CCN4 suppression would affect tumor cell growth, we conducted colony formation assays in LN299 cell lines harboring shRNA constructs aimed at suppressing endogenous CCN4 expression. These cell lines were chosen as they were amenable for colony formation assays. This glioblastoma cell line exhibits robust and reproducible clonogenic growth under adherent conditions. LN229 cells are widely used for clonogenic assays in glioblastoma studies evaluating gene perturbation or therapeutic effects [17,18,19]. Compared to the non-targeting shRNA control (shNT), knockdown of CCN4 with either of two independent shRNAs resulted in fewer colonies in vitro (Figure 4A). In this same system, re-expression of ectopic CCN4 lacking the 3′ UTR targeted by shRNA 70 could restore the growth phenotype (Figure 4B), demonstrating that the observed growth inhibitory effects were most likely caused by suppression of endogenous CCN4 expression. Similarly, cell viability and spherogenic growth were reduced upon shRNA-mediated knockdown of CCN4 in a patient-derived glioma stem-like cell, the GSC 6-27 (Figure 4C). We chose GSC 6-27 as it had the highest relative expression of CCN4 among our available GSC lines (Supplemental Figure S3), making it a suitable candidate for knockdown studies. Likewise shRNA-mediated knockdown of CCN4 resulted in increased latency and survival compared to control in mice orthotopically injected with GSC 6-27, *p*-value = 0.000052 (Figure 4D).

In order to see if CCN4 overexpression was affecting any growth promoting downstream signaling in alignment with the observed phenotype, we conducted a human phospho-kinase array using the GSC 7-11 line which was used for the screen. Overexpression of CCN4 resulted in phosphorylation of epidermal growth factor receptor (EGFR), Ephrin type-A receptor 6 (EphA6), and Ephrin type-B receptor 1(EphB1) when compared to GFP control (Supplemental Figure S4A). In U87MG overexpression of CCN4 also resulted in phosphorylation of EGFR (Supplemental Figure S4B). Of note, EGFR is a well-known driver in glioblastoma [15].

**Figure 5:**
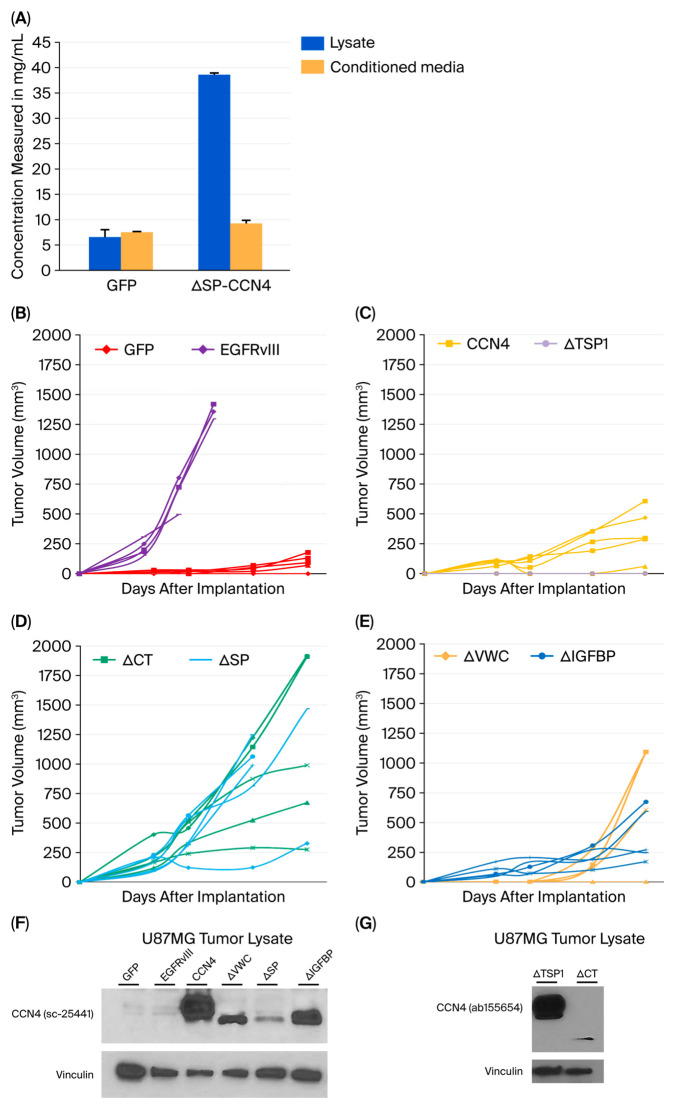
Structure-function analysis indicates that non-secreted ΔSP-CCN4 can drive growth while ΔTSP1-CCN4 fails to produce any tumors in vivo. (**A**) ELISA for CCN4 in cell lysate and conditioned medium from cells expressing ΔSP-CCN4 or GFP comparing the same amount of total protein from cell lysate and conditioned media for cells expressing either GFP or ΔSP-CCN4. Concentration measured in mg/mL. Bars represent standard error. Tumor growth after subcutaneous injection of 1.5 million U87MG cells expressing wild type or truncated CCN4, GFP, or EGFRvIII (n = 5 animals/group). Each line indicates the growth of an individual mouse. (**B**) GFP and EGFRvIII (**C**) CCN4 and ΔTSP1-CCN4 (**D**) ΔCT-CCN4 and ΔSP-CCN4 (**E**) ΔIGFBP-CCN4 and ΔVWC-CCN4 (**F**) Immunoblot analysis of tumor lysates using CCN4 sc-25441 which recognizes an antigen in the CT domain (**G**) Immunoblot analysis of tumor lysates using CCN4 ab155654 (epitope in VWC domain) to detect ΔCT-CCN4, which is not recognized by sc-25441.

To begin investigating the mechanisms through which CCN4 might be able to drive GBM growth, we conducted structural functional analysis. CCN4 is a secreted matricellular protein comprised of a signal peptide (SP) and four putative structural domains: Insulin-like Growth Factor Binding Protein-like Module (IGFBP), Von Willebrand Factor Type C Repeat (VWC), Thrombospondin Homology Type 1 Repeat (TSP1), and C-terminal Cysteine-knot Domain (CT) [20]. The signal peptide is a localization sequence that directs the protein to be secreted. In these experiments the established GBM xenograft model U87MG was used in which we induced ectopic expression of CCN4 constructs each lacking one of the domains mentioned above. We also transduced cells with vectors driving the expression of GFP, EGFRvIII or CCN4. Using ELISA we confirmed that overexpression of the ΔSP-CCN4 truncated isoform in the cells leads to overexpression of CCN4 in the lysate and not the media as a secreted protein (Figure 5A). U87MG was chosen for direct measurement of flank tumor growth after subcutaneous injection allowing for measured comparisons between overexpression of the various truncated isoforms. Surprisingly we found that ΔSP-CCN4 overexpression was able to drive tumor growth despite not being secreted (Figure 5C). Overexpression of other constructs were able to induce an acceleration of tumor growth as well with the exception of cells harboring the ΔTSP1 construct (Figure 5B–E). The TSP1 domain might either play a role in directly regulating a functional interaction or its absence might result in an overall destabilization of the protein tertiary structure. Immunoblots of the tumors confirmed overexpression of the respective truncated isoforms (Figure 5F,G).

## 3. Discussion

The dismal outcomes achieved with the current multimodal treatment strategies for GBM underscore the tremendous need for new effective therapies. Here we have leveraged TCGA datasets to conduct a functional genomics screen of prioritized ORFs that are overexpressed and/or amplified in GBM. To interrogate these potential drivers within a more relevant physiological context, the screening was accomplished in vivo in an orthotopic human-derived GSC model. Of the 12 pools, four of them demonstrated reduced latency. All four of these pools had ORFs with more than 2-fold enrichment except pool 1. We believe that this pool had reduced latency not because one particular ORF pushed towards faster growth, but rather there was some combined effect of these particular ORFs that made the pool have reduced latency without a defined ORF(s) showing enrichment. There were 4 other hits besides CCN4 which were PSCA, LYPD2, CHRAC1, and TATDN1. All of these genes are implicated in cancer with data either showing worse clinical features with higher expression or preclinical data as a growth promoter [21,22,23,24]. It is important to note that this screen was not exhaustive and only represented the 74 genes with the smallest nucleotide lengths. This indicates that there are potentially more ORF hits among overexpressed and/or amplified in GBM. Among the candidates from this screen, higher grade of glioma correlated with higher expression of CCN4 and CHRAC1 which was statistically significant. Patients with higher CCN4 expression had worse overall survival which was statistically significant while those with higher expression of CHRAC1 and PSCA demonstrated a trend toward worse overall survival. This further supports the strength of this screen in nominating potential growth promoters in glioblastoma. We hope that future studies can focus on the other candidates.

CCN4 emerged as a positive “hit” that was prioritized for further evaluation. As the fourth member of the CCN family, CCN4 is a secreted cysteine-rich matricellular protein considered to play a role in the development and progression of diverse disease processes such as cancer, fibrosis, metabolic and inflammatory disorders. Biologically, it has only been shown to interact with integrins and small leucine rich proteoglycans, such as decorin and biglycan [11]. Our functional analyses demonstrated that CCN4 overexpression drives tumor growth in multiple in vivo GBM models. Depletion of CCN4 markedly decreased colony formation in vitro with the growth phenotype restored upon ectopic expression of CCN4. Taken together, these data further support recent findings from other groups that CCN4 is a driver of GBM [12,13]. It is currently believed that CCN4 has both autocrine and paracrine signaling in glioblastoma where the autocrine signaling acts on α6β1 integrins to activate the FAK-AKT pathway while the paracrine signaling acts to maintain the density of M2 tumor associated macrophages [13]. In order to see if the brain-specific tumor microenvironment was necessary for the growth promoting phenotype of CCN4 overexpression that we observed, we conducted functional validation experiments in xenograft mouse flank injection models using established GBM lines. The phenotype was robust in these models in addition to the orthotopic GSC models. We interpret this to imply that perhaps the pro-tumorigenic effects of CCN4 are mediated more through autocrine signaling than through modulation of the brain-specific tumor microenvironment. A limitation of this study is that experiments were not performed in a blinded manner. However, the primary in vivo endpoints were based on objective measures, including survival with predefined humane endpoint criteria in orthotopic models, as well as caliper-based tumor measurements and predetermined maximum tumor size thresholds in flank xenografts. Nevertheless, observer bias cannot be completely excluded, and future studies incorporating blinded assessments are merited. Also, any translational interpretation of CCN4 as a potential biomarker or therapeutic target would be too preliminary as that would require integration with additional molecular, histological, and clinical variables, likely employing multivariate models and independent validation cohorts.

In the human phosphor-kinase array, overexpression of CCN4 resulted in phosphorylation of epidermal growth factor receptor (EGFR), Ephrin type-A receptor 6 (EphA6), and Ephrin type-B receptor 1(EphB1) when compared to GFP control. This is intriguing as it has not yet been shown to our knowledge that in glioblastoma overexpression of CCN4 is associated with EGFR phosphorylation. This is a preliminary mechanistic observation that may be an insight into another way that CCN4 can drive tumor growth. EphA6 phosphorylation may represent a compensatory, tumor restraining response to oncogenic EGFR signaling, potentially mediated through BMP pathway engagement or stress induced receptor crosstalk [25]. Although there are no published reports showing direct phosphorylation of EphB1 by EGFR in glioblastoma, it is well established that receptor tyrosine kinases can engage in signaling crosstalk. In several cancer types, EGFR and other RTKs have been shown to influence Eph receptor phosphorylation and downstream pathways either through shared downstream kinases or via physical RTK–RTK complexes. Thus, EGFR hyperactivation could plausibly modulate EphB1 signaling indirectly [26]. Also, both the GSC 7-11 line and the U87MG GBM line displayed EGFR phosphorylation with CCN4 overexpression implying that the downstream mechanism may be similar in both model types. However, these preliminary observations are hypothesis generating and lack functional validation through experiments perturbing the signaling in this context. More research behind the downstream signaling that enables CCN4 to drive glioblastoma is needed to further understand and validate these findings.

Toward a functional characterization of the role of CCN4 in GBM, we studied the differential effects of five domain-deleted isoforms compared with wild-type protein in an in vivo U87MG subcutaneous model. To our surprise, we found that overexpression of CCN4 lacking the signal peptide module (ΔSP-CCN4) resulted in a strong growth phenotype. This indicates that though CCN4 is thought to function as a secreted protein that interacts with integrins to promote growth, but one possibility is that it may be capable of promoting growth inside the cell however further work is needed to investigate this. In addition, we observed that overexpression of TSP1 domain-deleted CCN4 (ΔTSP1-CCN4) exerted a strong suppressive effect on tumor growth, resulting in no detectable tumor development. Presumably, the suppression of tumor growth by ΔTSP1-CCN4 could indicate a dominant negative effect that disrupted the required activity of endogenous CCN4, thus suggesting that the TSP1 domain is required for the growth promoting activity of CCN4 in GBM. Further research is needed to understand how CCN4 functions inside the cell and to explore the function of the TSP1 domain to promote tumor growth.

This work underscores the utility of the screening strategy that was employed. A number of studies have utilized various screening strategies in glioblastoma [27,28,29,30,31,32,33,34]. The methods range from CRISPR-Cas9 and short-hairpin RNA to PiggyBac Transposon. Only one of these studies employed ORFs for a gain-of-function overexpression screen but it was an in vitro screen and did not use GSC cells [28]. Most screens are loss-of-function screens which are designed to find vulnerabilities or tumor suppressors. One relative weakness of loss-of-function screens is that the hits are often essential housekeeping genes rather than unique targets for glioblastoma. An overexpression-based gain-of-function screening strategy is well suited to identify growth promoting targets. Overexpression can be done through ORFs as used here or through CRISPRa technology where instead of knocking out a gene there is targeted upregulation of endogenous genes [35] or PiggyBac technology [36]. Neither CRISPRa nor PiggyBac were employed in the glioblastoma screens cited here [27,28,29,30,31,32,33,34] but they are potential ways to design an overexpression gain-of-function screen. Our screening method has several advantages over CRISPRa or PiggyBac. With ORFs specific isoforms can be tested while in CRISPRa or PiggyBac only endogenous genes can be targeted for overexpression. Likewise, ORF overexpression can bypass epigenetic barriers whereas with CRISPRa or PiggyBac silenced promoters may not respond. The most sensitive screens will need robust phenotypes. ORF overexpression generates stronger phenotypes as the expression level can be higher while it is not as high for CRISPRa or PiggyBac. Overall, ORF overexpression screening is well suited for discovering novel drivers that are sufficient in the overexpression context to drive growth. This screening strategy can serve as a complimentary approach to the other screening methods described here. Despite its strengths it also has several weaknesses. For instance, there are limitations in library size and ORF nucleotide size while combination pooling effects can be difficult to deconvolute and interpret. Potentially, there may have been pools in this screen which had candidate ORFs that could not drive tumor growth faster due to combined effects of other overexpressed ORFs in the same pool masking it. Overall, we believe that this kind of ORF overexpression screen in a pooled orthotopic in vivo GSC model may serve as an additional platform for the discovery of more overexpressed drivers in glioblastoma and in various contexts such as after radiation or chemotherapy. It also presents itself as an opportunity to leverage genomic data sets for functional validation for the discovery of new potential targets that may one day advance the treatment of GBM.

## 4. Materials and Methods

### 4.1. Study Design

Tumor development in orthotopic mouse experiments was monitored periodically by bioluminescence imaging. Mice were watched closely for signs of tumor burden such as weight loss and changes in gait. Mice were sacrificed when tumor burden was assessed to be too excessive based on bioluminescence imaging and animal health. In these experiments sample sizes (n = 5 or greater per group) were sufficient to determine statically significant differences in survival between groups. For the in vitro colony formation and growth assays there were three or more biological replicates and at least three technical replicates. For all the studies conducted, the experimenter was not blinded.

### 4.2. Cell Culture

All cells were cultured at 37 °C in a humidified chamber with 5% CO_2_. GSCs were cultured in DMEM/F12 50/50 (Gibco, Waltham, MA, USA) supplemented with 1X B27 Supplement (Invitrogen, Carlsbad, CA, USA #17504-044), 20 ng/mL EGF (PeproTech, Cranbury, NJ, USA), 20 ng/mL bFGF (PeproTech), and 1% Pen/Strep. U87MG, LN340, and LN229 were cultured in DMEM (Gibco) supplemented with 10% FBS (Gibco) and 1% Pen/Strep (Gibco).

### 4.3. In Vitro Assays

Cell viability was measured using Cell Titer Glo 3D Cell Viability Assay (Promega, Madison, WI, USA) at various time points. For the colony formation assay, 1000 cells were seeded into each well in triplicate in a 6-well plate. After 10 to 15 days in culture, cells were fixed with 4% paraformaldehyde and stained with crystal violet.

### 4.4. Immunoblot and Human Phospho-Kinase Array

Protein lysates were electrophoresed by SDS-Page on 5–15% gradient polyacrylamide SDS gels and transferred to nitrocellulose membranes using a semi-dry transfer apparatus according to the manufacturer’s instructions (Bio-rad, Hercules, CA, USA). Membranes were blocked in 5% nonfat milk in TBST (10 mM Tris, pH 8.0, 150 mM NaCl, 0.5% Tween 20) for 60 min. After blocking, they were incubated in primary antibody diluted in 5% nonfat milk in TBST overnight at 4 degrees C. Membranes were then washed three times in TBST for 5 min each and incubated with a 1:5000 dilution of horseradish peroxidase-conjugated secondary antibodies in 5% nonfat milk in TBST for one hour at room temperature. Finally, membranes were washed with TBST three times and band detection was carried out by chemiluminescence reaction followed by film exposure. Phospho-kinase array was determined using the Proteome Profiler Human Phospho-Kinase Array Kit (R&D Systems, Minneapolis, MN, USA). Briefly, cells were lysed in RIPA lysis buffer. Lysates were centrifuged for 10 min at 16,900× *g* and 4 °C. Further analysis was performed according to the manufacturer’s protocol. Antibodies are listed in Table 1.

### 4.5. Plasmids

cDNA for all ORFs of interest from the Ultimate ORF collection (Invitrogen) were transferred by Gateway cloning into the bicistronic vector pHAGE-EF1α-IRES-GFP. pLKO shRNAs targeting CCN4 expression were purchased from SIGMA.

### 4.6. Screen

Genes overexpressed and/or amplified in GBM were selected from the TCGA Cell 2013 GBM datasets through cBioPortal [9] for use in the screening. mRNA expression z-scores relative to diploid samples with a z-score threshold of +/− 2.0 was used. The open reading frames (ORFs) with smallest nucleotide length were chosen building a list comprising 87 open reading frames, representing 74 genes. These 74 genes only represent the smallest 74 genes with 105 larger ones excluded from this screen. There were 12 pools with 5–8 ORFs in each pool. We used a multiplicity of infection (MOI) of 3. The ORFs were ordered based on their nucleotide size with pools assembled based on the smallest ORFs to larger ones. Each pool had ORFs that were similar in size to make sure the transduction efficiency of each ORF was similar in each pool.

### 4.7. Transduction

cDNA overexpression was accomplished via transduction of cells with fresh concentrated lentivirus. Medium was changed after 24 h, and by 72 h after transduction the cells were ready for any downstream application. In the case of the knockdown experiments, pLKO shRNA from fresh concentrated lentivirus was used to transduce cells in the presence of 8 µg/mL polybrene, grown for 48 h (medium was replaced after 24 h) and selected with 6 µg/mL of puromycin for six days. shRNA listed in Table 2.

### 4.8. Lentivirus Production

Lentiviral particles were generated in HEK293T cells by cotransfection of the transfer plasmid encoding the ORF, together with the envelope plasmid pMD2.G (Addgene #12259) and packaging plasmid psPAX2 (Addgene #12260) using Polyethylenimine (PEI) in Opti-MEM [37]. In the case of knockdown experiments, pLKO plasmid containing the shRNA insert was used together with the psPAX2 and pMD2.G in the same manner. Virus-containing supernatant was collected 72 h after transfection and filtered through a 0.45-µm filter (Corning, Corning, NY, USA). Ultracentrifugation at 23,000 rpm for 1.5 h at 4 °C was used to concentrate the virus.

### 4.9. Immunohistochemistry

Standard procedures were used to dehydrate and paraffin embed formalin-fixed tumors. Cut slices were rehydrated and the antigen was unmasked by heating at 95 °C for 30 min with an antigen unmasking solution (Citra Plus–Biogenex, Fremont, CA, USA). After baking and antigen unmasking, tumor samples were incubated in 3% hydrogen peroxide for 15 min, then blocked in a solution of 3% BSA, 10% goat serum, and 0.1% triton. Finally, samples were incubated in primary antibody, washed, incubated in HRP-conjugated secondary antibody, washed, and developed using DAB. Haematoxylin was used to counterstain.

### 4.10. Animal Studies

All animal manipulations were carried out in accordance with institutional, state, and federal laws under an approved protocol. Mice were anesthetized with intraperitoneal (IP) injections of ketamine (100 mg/kg)/xylazine. Experiments used of 4- to 6-week-old female nude mice purchased from the Department of Experimental Radiation Oncology, The University of Texas MD Anderson Cancer Center (Houston, TX, USA). A guide screw and a multiport microinfusion syringe pump (Harvard Apparatus, Holliston, MA, USA) were used for the orthotopic intracranial injections in a method previously described [38,39]. GSCs were used for all intracranial injections and 50,000 cells were injected per mouse. Intracranial tumors were monitored using a Xenogen IVIS-200 imaging system. For subcutaneous models, cells were injected in the flank. LN340 cells were mixed 1:1 with matrigel (Fisher, Waltham, MA, USA) before injection. Tumors were measured with a caliper and tumor volume was calculated using the standard formula: Volume = 0.5 × (l × w^2^). For experiments comparing GFP and CCN4 in U87MG and LN340, 3 million cells were injected per mouse. The number of animals used in each group was determined based on a priori power analysis (α = 0.05, power = 0.8) using estimates from preliminary data to detect a biologically relevant effect size. The minimum number of animals required to achieve statistical validity was used in accordance with the principles of the 3Rs (replacement, reduction, and mrefinement).

### 4.11. Validation

In addition to glioma stem-like cell lines GSC 7-11 and GSC 8-11 for validation we used GBM lines. Though glioma cell lines such as U87MG have limitations in fully recapitulating the molecular and cellular heterogeneity of glioblastoma, particularly due to long-term serum culture and divergence from the original tumor genotype our study was designed such that the initial discovery phase was performed in a patient-derived glioma stem cell (GSC) model (GSC 7-11), which more faithfully preserves the stem-like phenotype and molecular characteristics of primary glioblastoma. Following this screen, and validation in GSC 7-11 and GSC 8-11, we utilized established glioma cell lines, including U87MG and LN340, for mechanistic validation through overexpression studies [40,41]. This approach minimizes model-specific bias while leveraging the complementary strengths of each system.

### 4.12. Structural Functional Analysis

Constructs of truncated isoforms were verified via Sanger sequencing, and expression in U87MG cells was confirmed by immunoblotting. Tumor growth was measured after subcutaneous injection of 1.5 million U87MG cells expressing wild type or truncated CCN4, GFP, or EGFRvIII (n = 5 animals/group). Antibody ab155654 was used to detect ΔCT-CCN4, which is not recognized by sc-25441. Antibody sc-25441 recognizes an antigen in the CT domain, thus ab155654 (epitope in VWC domain) was used to detect the ΔCT-CCN4.

### 4.13. ELISA

The human CCN4 DuoSet ELISA kit (DY1627) from R&D systems was used to measure CCN4 concentrations in conditioned media and lysate according to manufacturer’s instructions.

### 4.14. Genomic DNA Extraction and qPCR

Frozen tumors derived from the GSCs that were orthotopically injected into the forebrains of nude mice were cut into small pieces using a sterile scalpel and resuspended in buffer P1 (Qiagen, Venlo, The Netherlands) supplemented with 100 µg/mL RNase A (Promega, USA). GentleMACS M tubes (Miltenyi Biotech, Bergisch Gladbach, Germany) and the gentleMACS dissociator (Miltenyi Biotech) were used to accomplish dissociation. Reference cells were not dissociated. A 1:20 volume:volume of SDS (Promega) was added to lyse cells, and they were incubated at room temperature for 30 min. Lysate was passed 20 times through a 22-gauge syringe needle. One volume of Phenol:Chloroform (Sigma Aldrich, St. Louis, MO, USA) was added to the lysate, after which it was vortexed and centrifuged at 12,000 rpm for 12 min. The upper phase was transferred to a sterile Eppendorf tube for a second extraction using one volume of chloroform (Sigma Aldrich). Again the upper phase was transferred to a sterile Eppendorf tube. Genomic DNA was precipitated using 0.1 volume of 3M NaCl (Sigma Aldrich) and 0.8 volumes of isopropanol (Fisher Scientific, Waltham, MA, USA), followed by centrifugation at 14,000 rpm for 1 h at 4 degrees C. The DNA pellet was washed in 70% ethanol (Fisher Scientific) and centrifuged again for 5 min at 14,000 rpm after vortexing. The DNA pellet was washed in ethanol, air-dried, and dissolved in UltraPure distilled water (Invitrogen, USA). DNA was quantified using a NanoDrop 2000 (Thermo Scientific, Waltham, MA, USA). qPCR was conducted using SYBR green (Sigma-Aldrich). The program consisted of dissociation for 2 min at 94 °C, and 40 amplification cycles of 15 s at 94 °C followed by 1 min at 60 °C. Calculations and analysis were performed using the comparative ΔΔCT method.

### 4.15. Computational Analysis

Analysis regarding RNA expression and glioma grade and survival for the candidate genes was done through data from the Chinese Glioma Genome Atlas (CGGA) [16]. Specifically, data was from their RNA-seq 325. RNA expression was Log2(FPKM + 1) where FPKM is fragments per kilobase of transcript per million mapped reads. For the survival analysis high expression was defined as above the median of that gene in the cohort while low expression was defined as below the median.

### 4.16. Statistical Analysis

Kaplan-Meier analysis was used for the survival studies. The LogRank (Mantel-Cox) test was used to test for statistical significance in the survival studies using GraphPad Prism 7 software. *p*-values less than 0.05 were considered to be statistically significant.

## Figures and Tables

**Table 1:** Antibodies.

Target	Vendor and Catalog Number	Application
CCN4	Santa Cruz Biotechnology sc-25441	WB, IHC
CCN4	Abcam ab155654	WB
CCN4	R&D Systems 1627-WS	ELISA, WB
Vinculin	Cell Signalling Technology 4650S	WB
GFAP	Abcam ab7260	IHC
Ki67	Abcam ab15580	IHC

**Table 2:** shRNA vectors.

shRNA	Vector Backbone	shRNA Target	Vendor	Catalog Number
sh70	pLKO	CCN4	Sigma	TRCN0000373970
sh91	pLKO	CCN4	Sigma	TRCN0000373891
sh50	pLKO	CCN4	Sigma	TRCN0000033350
sh51	pLKO	CCN4	Sigma	TRCN0000033351
sh52	pLKO	CCN4	Sigma	TRCN0000033352
shNT	pLKO	scrambled (negative control)	Addgene	Plasmid #1864

## Data Availability

Data from this study are included in this published article and its Supplementary Materials.

## Supplementary Materials

The following supporting information can be downloaded at: https://www.mdpi.com/article/10.3390/ijms27125227/s1.

## References

[1] Polivka J., Polivka J., Holubec L., Kubikova T., Priban V., Hes O., Pivovarcikova K., Treskova I. (2017). Advances in Experimental Targeted Therapy and Immunotherapy for Patients with Glioblastoma Multiforme. Anticancer Res..

[2] Dolecek T.A., Propp J.M., Stroup N.E., Kruchko C. (2012). CBTRUS statistical report: Primary brain and central nervous system tumors diagnosed in the United States in 2005–2009. Neuro Oncol..

[3] Louis D.N., Ohgaki H., Wiestler O.D., Cavenee W.K., Burger P.C., Jouvet A., Scheithauer B.W., Kleihues P. (2007). The 2007 WHO classification of tumours of the central nervous system. Acta Neuropathol..

[4] Ostrom Q.T., Gittleman H., Liao P., Rouse C., Chen Y., Dowling J., Wolinsky Y., Kruchko C., Barnholtz-Sloan J. (2014). CBTRUS statistical report: Primary brain and central nervous system tumors diagnosed in the United States in 2007–2011. Neuro Oncol..

[5] Polivka J., Polivka J., Rohan V., Topolcan O., Ferda J. (2012). New molecularly targeted therapies for glioblastoma multiforme. Anticancer Res..

[6] Furnari F.B., Fenton T., Bachoo R.M., Mukasa A., Stommel J.M., Stegh A., Hahn W.C., Ligon K.L., Louis D.N., Brennan C. (2007). Malignant astrocytic glioma: Genetics, biology, and paths to treatment. Genes Dev..

[7] Krex D., Klink B., Hartmann C., von Deimling A., Pietsch T., Simon M., Sabel M., Steinbach J.P., Heese O., Reifenberger G. (2007). Long-term survival with glioblastoma multiforme. Brain J. Neurol..

[8] The Cancer Genome Atlas Research Network (2008). Comprehensive genomic characterization defines human glioblastoma genes and core pathways. Nature.

[9] Brennan C.W., Verhaak R.G., McKenna A., Campos B., Noushmehr H., Salama S.R., Zheng S., Chakravarty D., Sanborn J.Z., Berman S.H. (2013). The Somatic Genomic Landscape of Glioblastoma. Cell.

[10] Bhat K.P.L., Balasubramaniyan V., Vaillant B., Ezhilarasan R., Hummelink K., Hollingsworth F., Wani K., Heathcock L., James J.D., Goodman L.D. (2013). Mesenchymal differentiation mediated by NF-κB promotes radiation resistance in glioblastoma. Cancer Cell.

[11] Singh K., Oladipupo S.S. (2024). An overview of CCN4 (WISP1) role in human diseases. J. Transl. Med..

[12] Jing D., Zhang Q., Yu H., Zhao Y., Shen L. (2017). Identification of WISP1 as a novel oncogene in glioblastoma. Int. J. Oncol..

[13] Tao W., Chu C., Zhou W., Huang Z., Zhai K., Fang X., Huang Q., Zhang A., Wang X., Yu X. (2020). Dual Role of WISP1 in maintaining glioma stem cells and tumor-supportive macrophages in glioblastoma. Nat. Commun..

[14] Thomas J.G., Kerrigan B.C.P., Hossain A., Gumin J., Shinojima N., Nwajei F., Ezhilarasan R., Love P., Sulman E.P., Lang F.F. (2017). Ionizing radiation augments glioma tropism of mesenchymal stem cells. J. Neurosurg..

[15] Nathanson D.A., Gini B., Mottahedeh J., Visnyei K., Koga T., Gomez G., Eskin A., Hwang K., Wang J., Masui K. (2014). Targeted therapy resistance mediated by dynamic regulation of extrachromosomal mutant EGFR DNA. Science.

[16] Zhao Z., Zhang K.N., Wang Q., Li G., Zeng F., Zhang Y., Wu F., Chai R., Wang Z., Zhang C. (2021). Chinese Glioma Genome Atlas (CGGA): A Comprehensive Resource with Functional Genomic Data from Chinese Glioma Patients. Genom. Proteom. Bioinform..

[17] Zhao W., Gao D., Ning L., Jiang Y., Li Z., Huang B., Chen A., Wang C., Liu Y. (2022). Prodigiosin inhibits the proliferation of glioblastoma by regulating the KIAA1524/PP2A signaling pathway. Sci. Rep..

[18] Chu F., Wu P., Mu M., Hu S., Niu C. (2023). MGCG regulates glioblastoma tumorigenicity via hnRNPK/ATG2A and promotes autophagy. Cell Death Dis..

[19] Wu C., Shen Y., Shi L., Zhang J., Guo T., Zhou L., Wang W., Zhang X., Yu R., Liu X. (2023). UBA1 inhibition contributes radiosensitization of glioblastoma cells *via* blocking DNA damage repair. Front. Pharmacol..

[20] Nivison M.P., Meier K.E. (2018). The role of CCN4/WISP-1 in the cancerous phenotype. Cancer Manag. Res..

[21] Zhigang Z., Wenlv S. (2004). Prostate stem cell antigen (PSCA) expression in human prostate cancer tissues and its potential role in prostate carcinogenesis and progression of prostate cancer. World J. Surg. Oncol..

[22] Russ E., Bhuvaneshwar K., Wang G., Jin B., Gage M.M., Madhavan S., Gusev Y., Upadhyay G. (2021). High mRNA expression of LY6 gene family is associated with overall survival outcome in pancreatic ductal adenocarcinoma. Oncotarget.

[23] Wang M., Li S., Guo W., Wang L., Huang J., Zhuo J., Lai B., Liao C., Ge T., Nie Y. (2022). CHRAC1 promotes human lung cancer growth through regulating YAP transcriptional activity. Carcinogenesis.

[24] Niu Z., Zhang X., Li W., Ming Z., Zhong Y., Hou Y., Zhang Y., Meng X., Wang W., Deng W. (2016). The role and potential mechanisms of LncRNA-TATDN1 on metastasis and invasion of non-small cell lung cancer. Oncotarget.

[25] Raja E., Morikawa M., Nishida J., Tanabe R., Takahashi K., Seeherman H.J., Saito N., Todo T., Miyazono K. (2019). Tyrosine kinase Eph receptor A6 sensitizes glioma-initiating cells towards bone morphogenetic protein-induced apoptosis. Cancer Sci..

[26] Franco Nitta C., Green E.W., Jhamba E.D., Keth J.M., Ortiz-Caraveo I., Grattan R.M., Schodt D.J., Gibson A.C., Rajput A., Lidke K.A. (2021). EGFR transactivates RON to drive oncogenic crosstalk. eLife.

[27] Chow R.D., Guzman C.D., Wang G., Schmidt F., Youngblood M.W., Ye L., Errami Y., Dong M.B., Martinez M.A., Zhang S. (2017). AAV-mediated direct in vivo CRISPR screen identifies functional suppressors in glioblastoma. Nat. Neurosci..

[28] Quayle S.N., Chheda M.G., Shukla S.A., Wiedemeyer R., Tamayo P., Dewan R.W., Zhuang L., Huang-Hobbs E., Haidar S., Xiao Y. (2012). Integrative functional genomics identifies RINT1 as a novel GBM oncogene. Neuro Oncol..

[29] Puca F., Yu F., Bartolacci C., Pettazzoni P., Carugo A., Huang-Hobbs E., Liu J., Zanca C., Carbone F., Del Poggetto E. (2021). Medium-Chain Acyl-CoA Dehydrogenase Protects Mitochondria from Lipid Peroxidation in Glioblastoma. Cancer Discov..

[30] Noorani I., de la Rosa J., Choi Y.H., Strong A., Ponstingl H., Vijayabaskar M.S., Lee J., Lee E., Richard-Londt A., Friedrich M. (2020). Correction to: PiggyBac mutagenesis and exome sequencing identify genetic driver landscapes and potential therapeutic targets of EGFR-mutant gliomas. Genome Biol..

[31] Weishaupt H., Čančer M., Rosén G., Holmberg K.O., Häggqvist S., Bunikis I., Jiang Y., Sreedharan S., Gyllensten U., Becher O.J. (2023). Novel cancer gene discovery using a forward genetic screen in RCAS-PDGFB-driven gliomas. Neuro Oncol..

[32] MacLeod G., Bozek D.A., Rajakulendran N., Monteiro V., Ahmadi M., Steinhart Z., Kushida M.M., Yu H., Coutinho F.J., Cavalli F.M.G. (2019). Genome-Wide CRISPR-Cas9 Screens Expose Genetic Vulnerabilities and Mechanisms of Temozolomide Sensitivity in Glioblastoma Stem Cells. Cell Rep..

[33] Li X., Zhang W., Fang Y., Sun T., Chen J., Tian R. (2025). Large-scale CRISPRi screens link metabolic stress to glioblastoma chemoresistance. J. Transl. Med..

[34] Savage N., Danis E., Chokshi C.R., Custers S., Shaikh M.V., Miletic P., Venugopal C., Brown K.R., Vibhakar R., Moffat J. (2025). CRISPR screen reveals SOX2 as a critical regulator of CD133 and cellular stress response in glioblastoma. Sci. Rep..

[35] Gilbert L.A., Horlbeck M.A., Adamson B., Villalta J.E., Chen Y., Whitehead E.H., Guimaraes C., Panning B., Ploegh H.L., Bassik M.C. (2014). Genome-Scale CRISPR-Mediated Control of Gene Repression and Activation. Cell.

[36] Chen L., Stuart L., Ohsumi T.K., Burgess S., Varshney G.K., Dastur A., Borowsky M., Benes C., Lacy-Hulbert A., Schmidt E.V. (2013). Transposon activation mutagenesis as a screening tool for identifying resistance to cancer therapeutics. BMC Cancer.

[37] Tang Y., Garson K., Li L., Vanderhyden B.C. (2015). Optimization of lentiviral vector production using polyethylenimine-mediated transfection. Oncol. Lett..

[38] Nakamizo A., Marini F., Amano T., Khan A., Studeny M., Gumin J., Chen J., Hentschel S., Vecil G., Dembinski J. (2005). Human bone marrow-derived mesenchymal stem cells in the treatment of gliomas. Cancer Res..

[39] Lal S., Lacroix M., Tofilon P., Fuller G.N., Sawaya R., Lang F.F. (2000). An implantable guide-screw system for brain tumor studies in small animals. J. Neurosurg..

[40] Ramakrishnan V., Xu B., Akers J., Nguyen T., Ma J., Dhawan S., Ning J., Mao Y., Hua W., Kokkoli E. (2020). Radiation-induced extracellular vesicle (EV) release of miR-603 promotes IGF1-mediated stem cell state in glioblastomas. eBioMedicine.

[41] Allen M., Bjerke M., Edlund H., Nelander S., Westermark B. (2016). Origin of the U87MG glioma cell line: Good news and bad news. Sci. Transl. Med..

