# Supplementary figures and images for: In Vivo ORF Overexpression Screening Identifies CCN4 as a Regulator of Glioblastoma Growth Validated Across Multiple Models
Source: Int J Mol Sci. 2026 Jun 9;27(12):5227. doi: 10.3390/ijms27125227 (PMC13299954; doi:10.3390/ijms27125227)

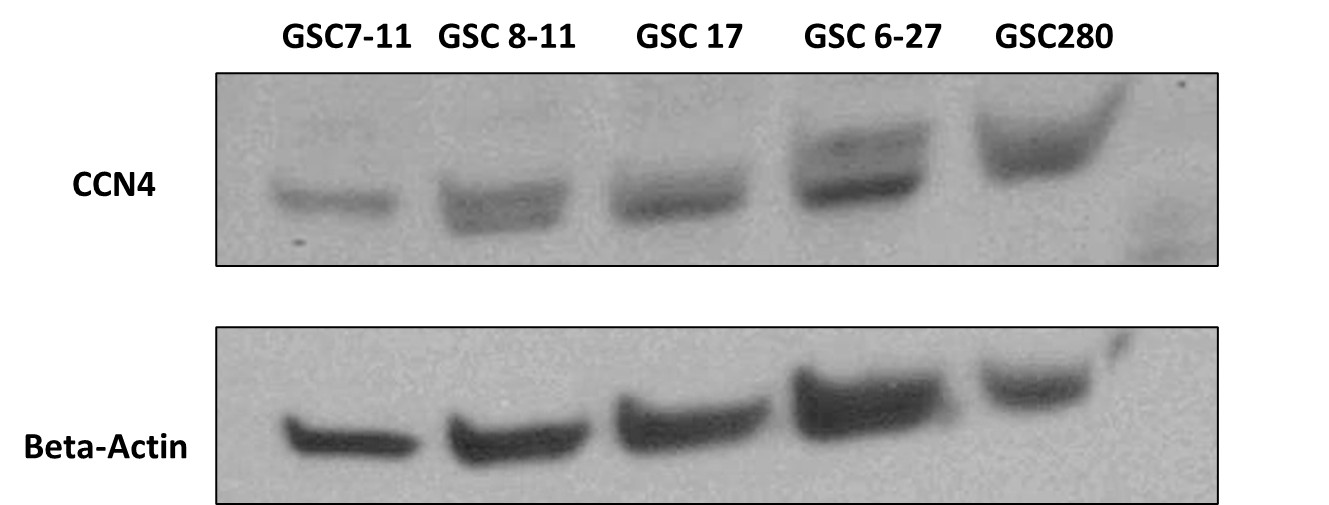

Supplement: Supplementary file 1 [file ijms-27-05227-s001.zip › Supplemental Figure 3.jpg]

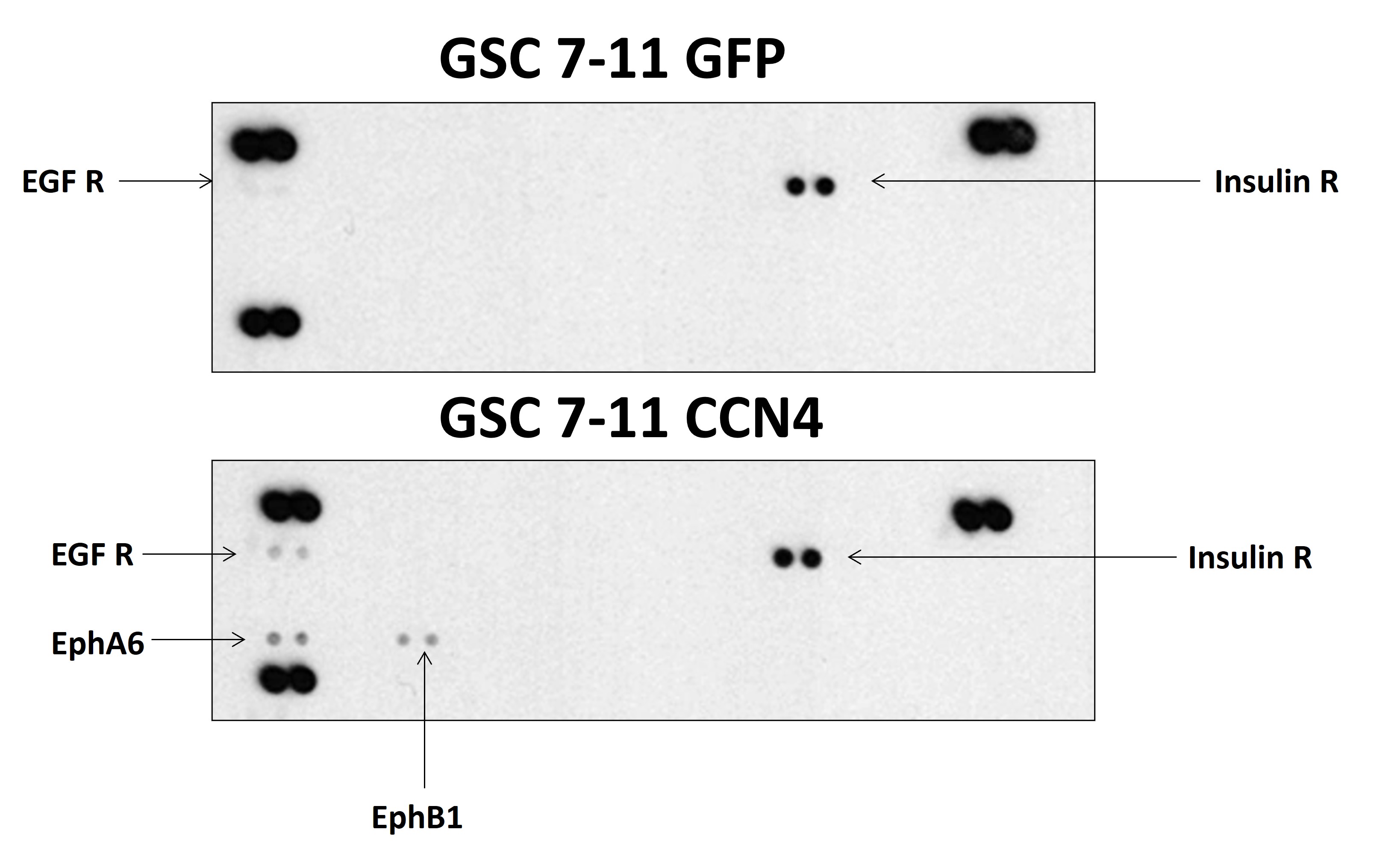

Supplement: Supplementary file 1 [file ijms-27-05227-s001.zip › Supplemental Figure 4A.jpg]

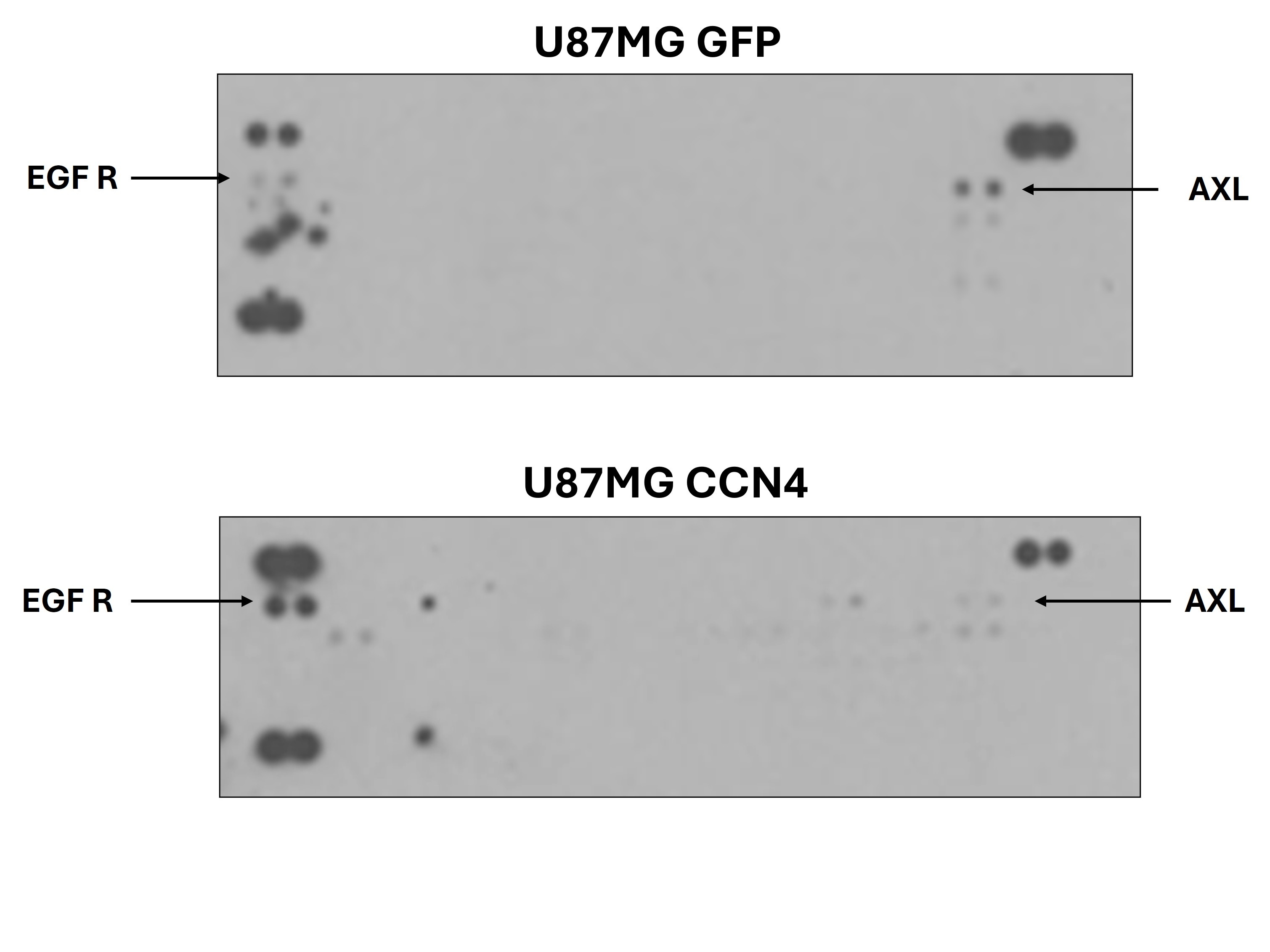

Supplement: Supplementary file 1 [file ijms-27-05227-s001.zip › Supplemental Figure 4B.jpg]

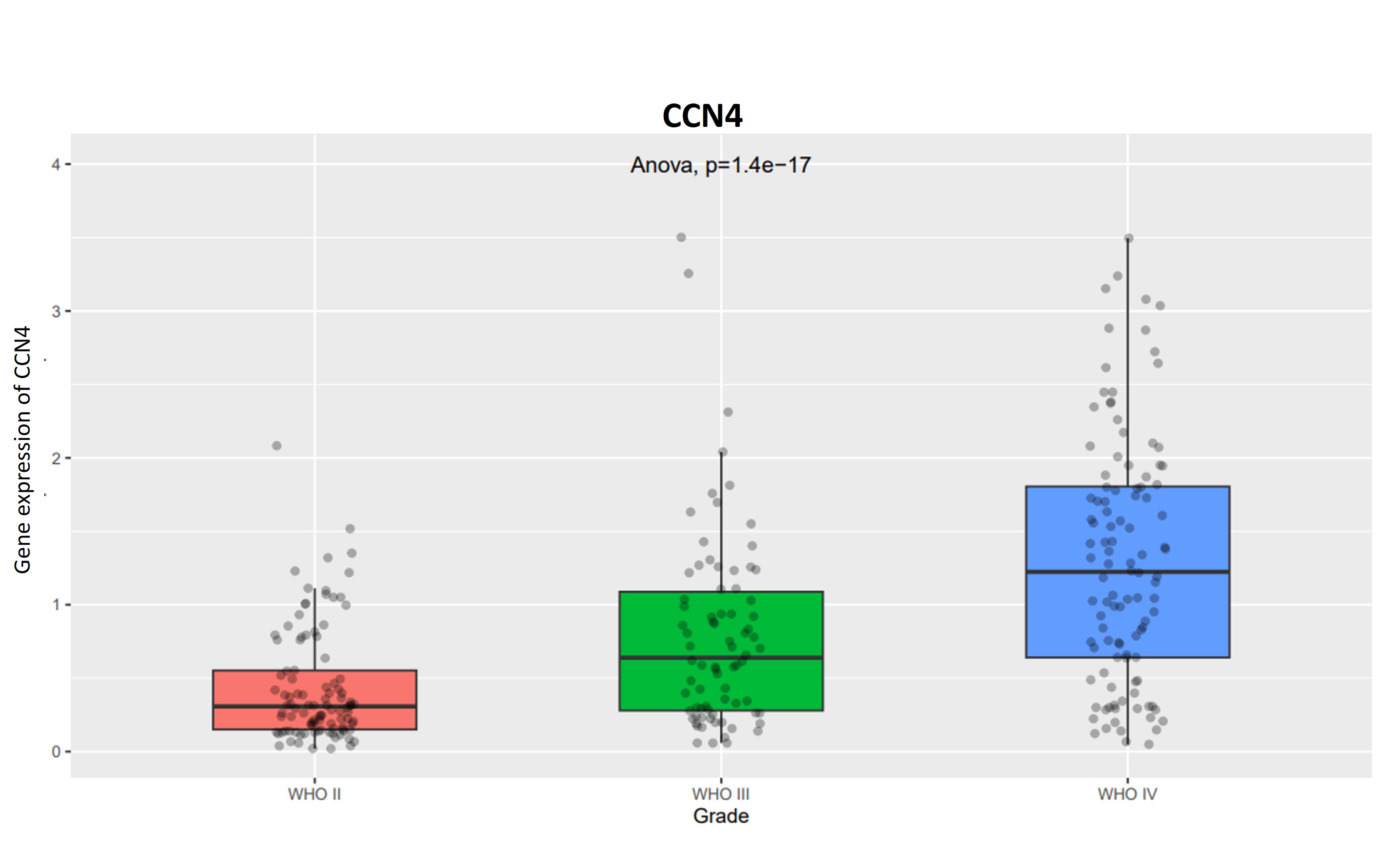

Supplement: Supplementary file 1 [file ijms-27-05227-s001.zip › Supplemental Figure 1A.jpg]

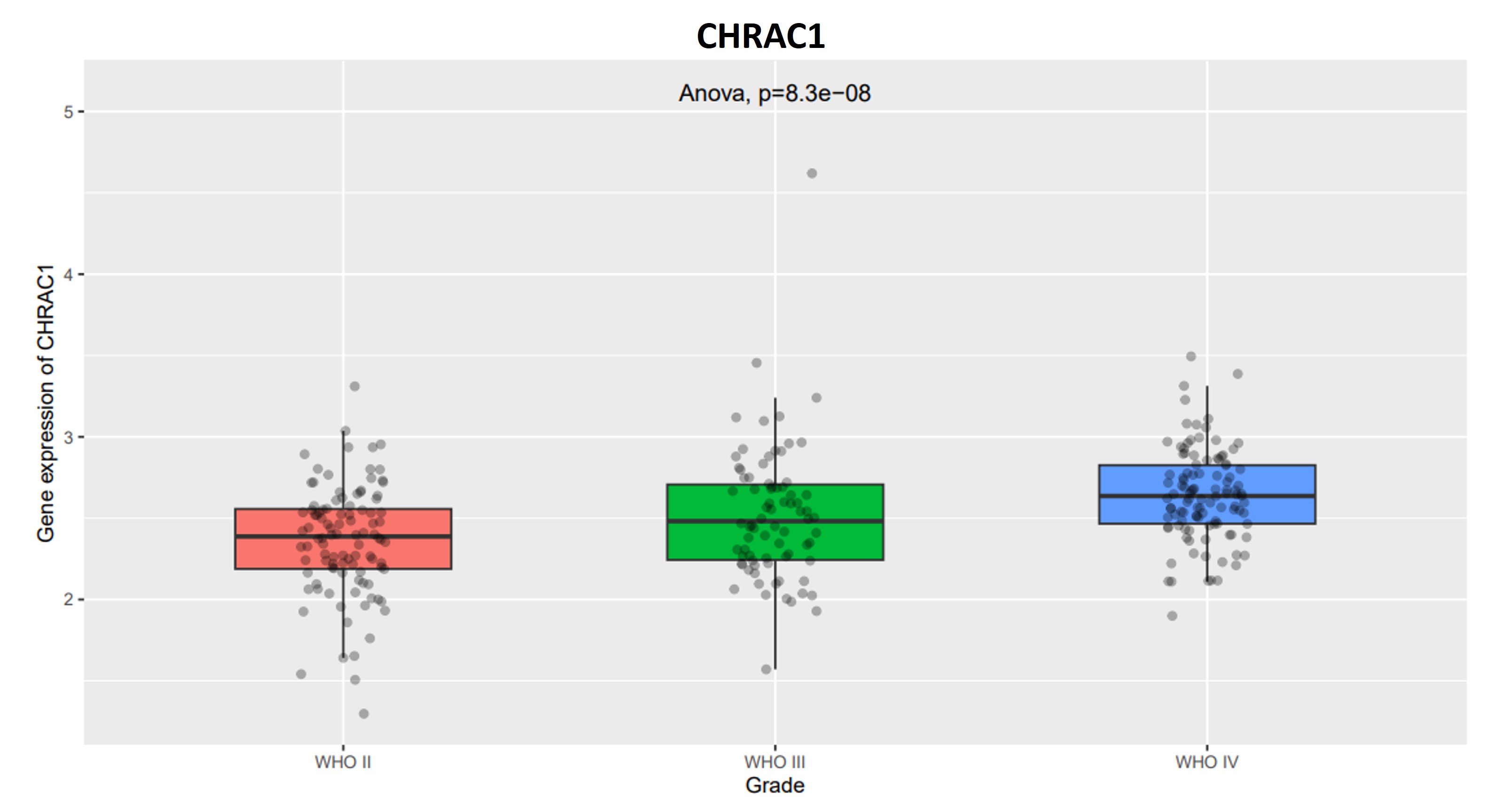

Supplement: Supplementary file 1 [file ijms-27-05227-s001.zip › Supplemental Figure 1B.jpg]

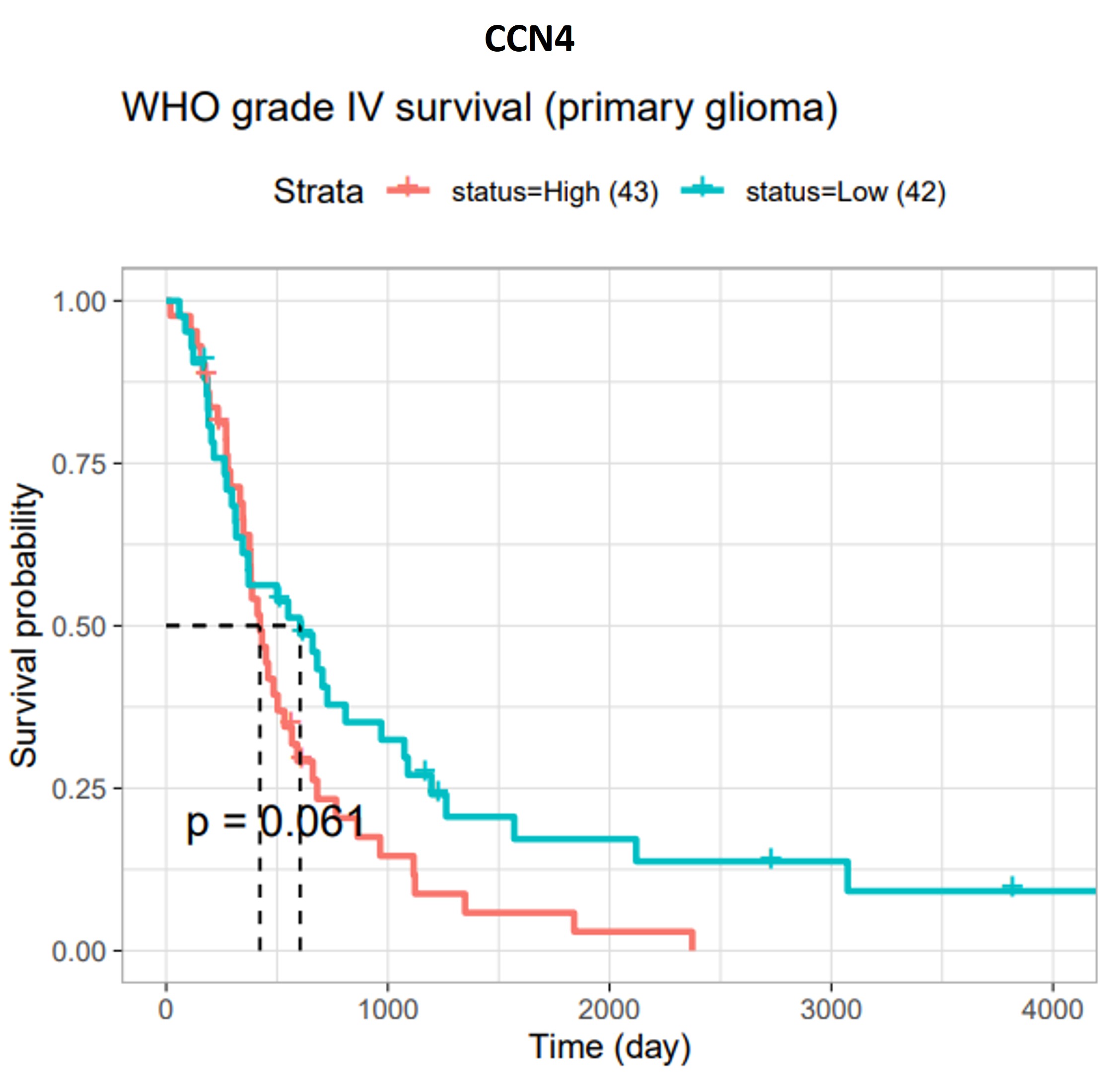

Supplement: Supplementary file 1 [file ijms-27-05227-s001.zip › Supplemental Figure 1C.jpg]

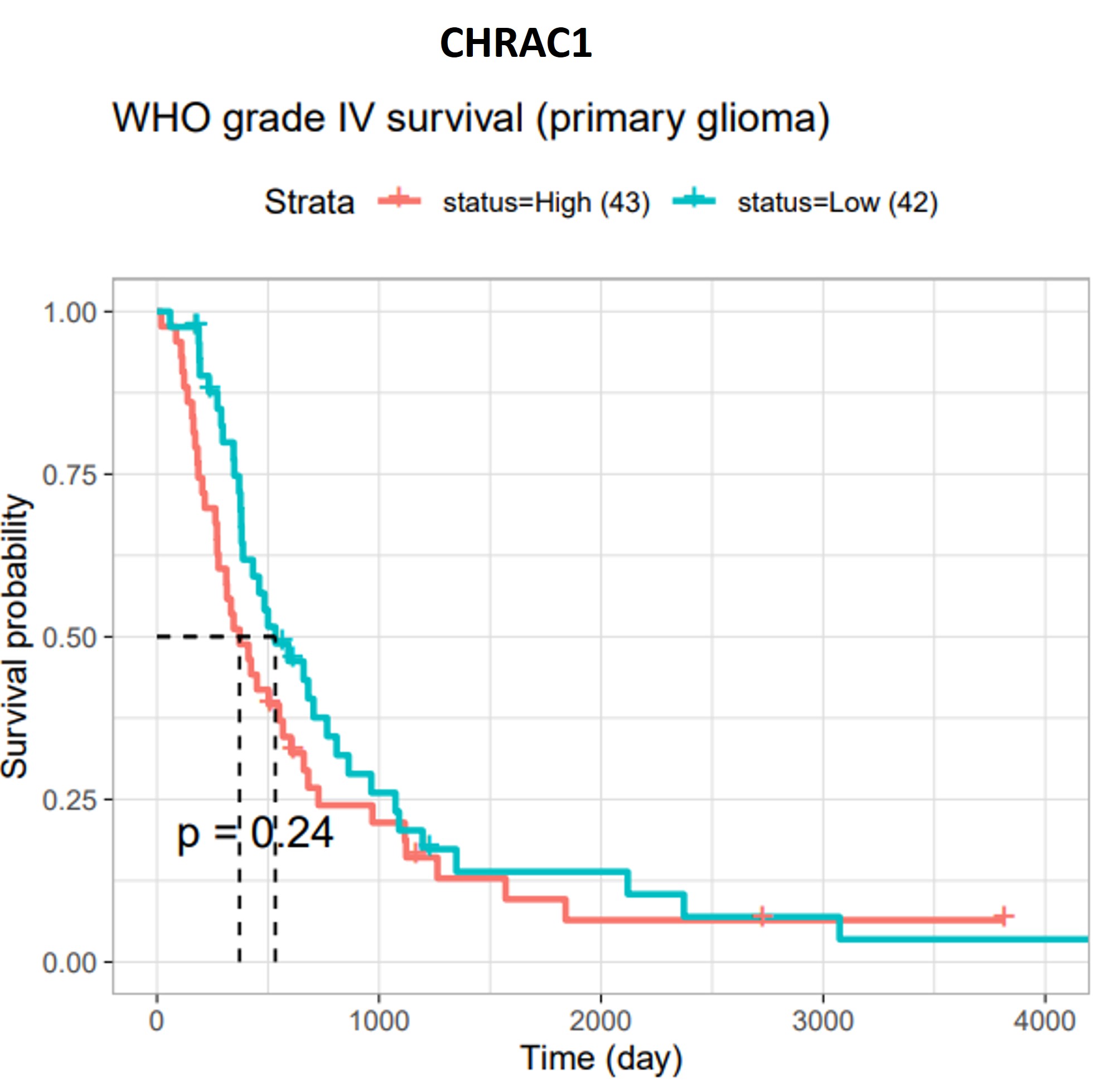

Supplement: Supplementary file 1 [file ijms-27-05227-s001.zip › Supplemental Figure 1D.jpg]

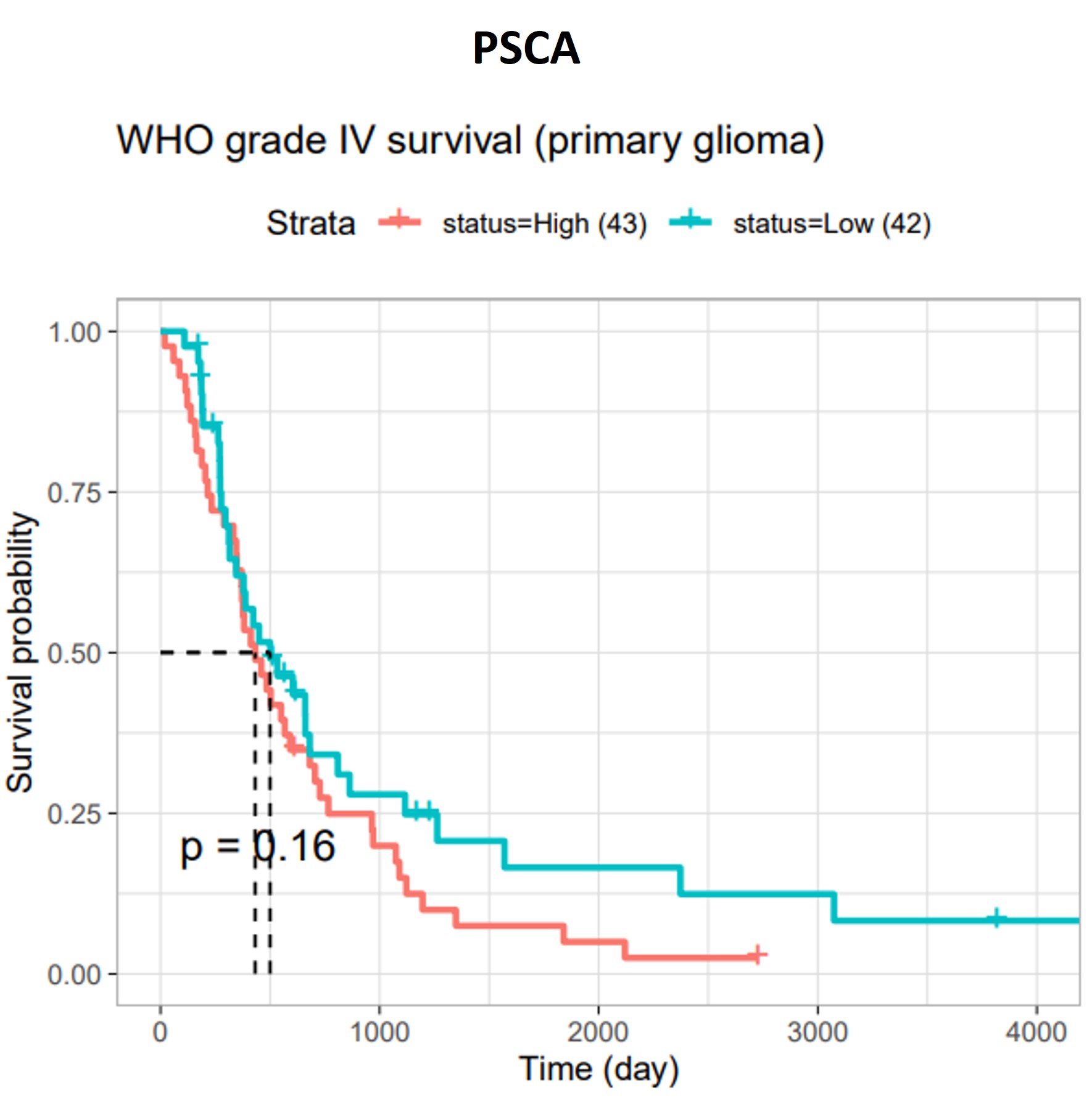

Supplement: Supplementary file 1 [file ijms-27-05227-s001.zip › Supplemental Figure 1E.jpg]

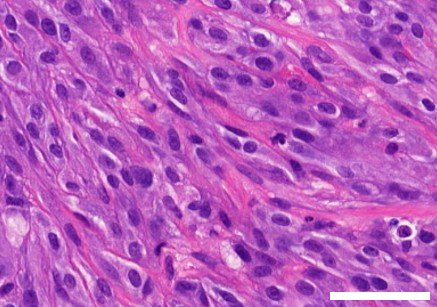

Supplement: Supplementary file 1 [file ijms-27-05227-s001.zip › Supplemental Figure 2.jpg]
